# Can self-validating neuroenhancement be autonomous?

**DOI:** 10.1007/s11019-019-09905-7

**Published:** 2019-05-29

**Authors:** Jukka Varelius

**Affiliations:** Department of Philosophy, Contemporary History, and Political Science, University of Turku, 20014 Turku, Finland

**Keywords:** Autonomy, Human enhancement, Neuroscience, Personality change

## Abstract

Consider that an individual improves her capacities by neuroscientific means. It turns out that, besides altering her in the way(s) she intended, the enhancement also changes her personality in significant way(s) she did not foresee. Yet the person endorses her new self because the neuroenhancement she underwent changed her. Can the person’s approval of her new personality be autonomous? While questions of autonomy have already gathered a significant amount of attention in philosophical literature on human enhancement, the problem just described—henceforth referred to as the question whether self-validating neuroenhancement can be autonomous—would not appear to have received due consideration. This article takes a step towards remedying the shortage. I start by explicating the main points of departure of its argument. In the subsequent sections of the article, I consider several possible reasons for deeming self-validating neuroenhancement incompatible with autonomy. On the basis of the consideration, I propose that self-validating neuroenhancement can be autonomous.

## Introduction

Kelly wanted to improve herself. After considering different ways of doing that, she decided to use novel neuroscientific means. Just as she desired, she became more creative, cognitively able, and cheerful than she was before. Not radically, but still significantly. However, before her enhancement process Kelly thought that, the improvements notwithstanding, she would ultimately remain the same old Kelly. She would be a more creative art director, a quicker thinking businesswoman, and have some more laughs, but basically still be the Kelly she has always been. But after her enhancement, Kelly sees the world and herself differently than she used to do and wants to behave in ways she did not expect to want. While Kelly is quite pleased with her new personality, her spouse, Larry, who was always somewhat suspicious about Kelly’s self-improvement project, thinks that Kelly approves of her new self just because her neuroenhancement changed her. In Larry’s view, the enhancement process Kelly underwent did not only alter her personality, but also made her endorse her new self. Accordingly, Larry maintains that Kelly’s enhancement process was not autonomous because it validated itself.

Autonomy plays an important role in common morality and in moral philosophy (see, e.g., Beauchamp and Childress 2013) and questions related to autonomy have already gathered a significant amount of attention in the moral philosophical literature on human enhancement (see, e.g., Heilinger and Crone 2014; Juth 2011; Pömsl and Friedrich 2017; Schaefer et al. 2014). Yet whether self-validating neuroenhancement—neuroenhancement in which an individual’s endorsement of her new self is caused by the enhancement process she underwent—can be autonomous would not appear to have received due consideration.^1^ If it is incompatible with autonomy that would apparently be a good reason to avoid neuroenhancement likely to be self-validating, at least when other things are being equal.^2^ This motivates the following reflections. I start by explicating the main starting points of the argument of this article. In the three subsequent sections, I consider several possible reasons for deeming self-validating neuroenhancement incompatible with individual autonomy. On the basis of the consideration, I conclude that self-validating neuroenhancement can be autonomous.

## Main starting points

Contemporary philosophical debate on human enhancement has mainly focused on improvement of mood, cognition, morality, and physical abilities and expansion of lifespan with techniques enabled by novel or anticipated results from neuroscience and genetics (see, e.g., Levy 2007; Meulen et al. 2017; Savulescu and Bostrom 2009; Schermer 2017). Instead of going into the problems related to how, precisely, human enhancement should be defined (cf., e.g., Raus et al. 2014; Savulescu et al. 2011; Wolpe 2002), I here settle for the following rough characterization of *neuroenhancement* of humans: human neuroenhancement refers to improving the capacities of, or producing new capacities for, healthy individuals by means provided by neuroscience.^3^ In self-validating neuroenhancement, the enhanced individual’s personality changes in significant way(s) she did not foresee and the individual endorses her new self because the neurointervention changed her. Here the individual’s acceptance of her new personality is thus caused by the neuroenhancement she underwent. By the individual’s new personality—now used interchangeably with her new self,—I thus refer to the novel personality the individual acquires within the enhancement process she undergoes. Correspondingly, by talking about an enhanced individual’s old personality and about her old self, I refer to the personality she had when she decided to undergo the neuroenhancement process she undergoes. I understand personality in the colloquial sense in which the term denotes the combination of characteristics or qualities that form an individual’s distinctive character and to endorse in the colloquial sense in which to endorse something means to accept it, to approve of it. I focus on neuroenhancement of mental capacities.^4^

Consider a case that is otherwise similar to Kelly’s except that the individual improved in it, Mike, was aware of all of the ways in which his enhancement changed his personality already before the enhancement process started. His foreknowledge of the alterations notwithstanding, Mike’s current endorsement of his new self could be taken to result from his having been changed by neuroscientific means.^5^ Yet that it involves unforeseen consequences is one of the central concerns neuroenhancement raises. While some initially unpredicted consequences might be immaterial, the acquisition of a new personality would most plausibly usually be important. Accordingly, accounting for such a consequence is arguably, if not evidently, relevant in assessing neuroenhancement. This motivates my understanding self-validating neuroenhancement in the above characterized way. Yet the considerations presented below pertain to assessing the autonomy of the kind of fully informed self-validating neuroenhancement just characterized as well.

That a person enhanced by neuroscientific means endorses the new personality she may acquire within her enhancement process is, of course, not the only possibility. In self-invalidating neuroenhancement, the enhanced person rejects his new self because the neurointervention changed him. In indecisive neuroenhancement, the enhanced person remains undecided whether to endorse or to reject her new personality because the neurointervention changed her. While the following considerations apply, *mutatis mutandis*, to assessing the autonomy of self-invalidating and indecisive neuroenhancement as well, for the sake of simplicity I now focus on self-validating neuroenhancement.^6^

Insofar as neuroscientific means by which to effectively enhance human capacities do not exist (see, e.g., Meulen et al. 2017, see Sect. “Main starting points”; Schleim and Quednow 2018), the idea of self-validating neuroenhancement is merely theoretical. However, given the interest in the development of neuroscientific technology (see, e.g., Marsh 2018) and the apparently widespread appeal of self-improvement, I assume that the prospect that such means become available is worth preparing for already. Especially to the extent that neuroscientific means of enhancement are hypothetical, determining the precise mechanisms by which they would improve human capacities is difficult. Yet it appears justifiable to assume that in practice neuroenhancement of mental capacities would work by affecting brain and other relevant bodily mechanisms by such means as drugs and electric devices (see also Sect. “Is the main reason for considering self-validating neuroenhancement heteronomous plausible?”).

In their influential bioethical work, Beauchamp and Childress (2013, pp. 101–102) characterize individual autonomy as follows:

At a minimum, personal autonomy encompasses self-rule that is free from both controlling interference by others and limitations that prevent meaningful choice, such as inadequate understanding. The autonomous individual acts freely in accordance with a self-chosen plan, analogous to the way an independent government manages its territories and sets its policies. In contrast, a person of diminished autonomy is in some material respect controlled by others or incapable of deliberating or acting on the basis of his or her desires and plans. For example, cognitively challenged individuals and prisoners often have diminished autonomy. Mental incapacitation limits the autonomy of a person with severe mental handicap, whereas coercive institutionalization constrains a prisoner’s autonomy.

While the understanding of autonomy has not gone without criticism (see, e.g., Dive and Newson 2018; Manson and O’Neill 2007),^7^ it nevertheless continues to play a central role in contemporary bioethics.^8^ Whether Kelly’s self-validating neuroenhancement meets its criteria of free, intentional, and knowing choice can also be of interest even to those who understand autonomy in different terms.^9^ Accordingly, I here employ the just described conception of individual autonomy.

It might be taken that an individual’s endorsement of the new personality she acquires within self-validating neuroenhancement is not problematic in terms of autonomy insofar as the acceptance does not employ mental capacities affected by the neuroenhancement process she underwent. Yet in connection with enhancement that affects a person as extensively as self-validating neuroenhancement does an agent’s endorsement of her new personality may not employ capacities unaffected by the neurointervention at all. It would also appear to be difficult to determine to what extent, if any, an agent’s approval of her new personality would employ capacities unaffected by the neuroenhancement process she underwent. The question whether self-validating neuroenhancement can be autonomous is evidently not the only problem relating to (self-validating) neuroenhancement. Yet, for reasons of space, I here abstract from the other difficulties (self-validating) neuroenhancement faces.^10^

## Why would self-validating neuroenhancement be incompatible with autonomy?

It has been proposed that the availability of the kind of techniques focused on in philosophical literature on neuroenhancement would eradicate all grounds for choosing in one way rather than another. In this view, if our mental life became as radically modifiable as the techniques are seen to make it, also the grounds of one’s choices could always be altered with the technology. Given that all choices would ultimately become arbitrary in that sense, autonomy would be lost (cf., e.g., Owens 2007). However, as a matter of psychological fact, the claim that the novel human enhancement techniques would destroy all grounds for choosing in one way rather than another appears simply false. At least when other things are being equal, that a person could always modify herself to want something different than what she in fact wants does not entail that she wants to modify herself in that way or that she has reason to do so. Many ways of altering oneself can be quite irrelevant to one, some people would assumedly reject them all (see also, e.g., Danaher 2014). Accordingly, the mere fact that Kelly would have the possibility to change herself in some further way(s) than she did plausibly does not mean that her endorsement of her new self cannot be autonomous.

But, as proposed, at the stage of deciding to enhance herself by neuroscientific means, Kelly did not foresee that after the process she will not be the same old Kelly anymore. Given that autonomy presupposes that a person is adequately informed, it would therefore seem that Kelly’s initial decision to engage in the neuroenhancement process she underwent was not autonomous. And it might now be taken that the lack of autonomy related to her initial decision to enhance herself with neuroscientific means entails that Kelly’s current approval of her new personality cannot be autonomous either. Now, making a self-governing choice whether or not to undergo the kind of enhancement process Kelly underwent would indeed seem to presuppose that a person is at least aware of the possibility that the process may change her in some unexpected way(s). However, after her enhancement process Kelly is well aware both of the possibility that the process can alter her in some initially unforeseen way(s) and of the precise ways in which it actually did change her. Accordingly, Kelly’s current endorsement of her new personality does not suffer from the kind of lack of information that compromised the autonomy of her initial decision to enhance herself by neuroscientific means.^11^

Several authors have, however, been concerned about the way neuroscientific modification of mental capacities is seen to work. Instead of employing the rational abilities of the individuals whose capacities are modified with neuroscientific techniques, neurointerventions are perceived to function by directly affecting the neural underpinnings of the capacities altered with the techniques (cf., e.g., Levy 2007, Chap. 3). Witt expresses the worry related to this feature of neurointerventions—henceforth referred to as the directness of neuroscientific techniques—in connection with a patient whose treatment caused a personality change the patient endorsed because the therapy changed him. Witt (2017, p. 388, emphasis in original) writes as follows:

The stimulation [the treatment in question] not only altered his [the patient’s] personality and his central projects, but also made him approve of those changes. If this is what happens, there is a relevant sense in which the approval is not his *own*: like the change itself, it is not under his control. Instead of being the result of rational, independent deliberation on his side, it has been brought about by the intervention. Therefore, it counts as heteronomous and to that extent unsuitable for informed consent.^12^

If the directness of neurointerventions compromised autonomy, the problem would apparently concern the use of neurointerventions in general, not only self-validating neuroenhancement.^13^ Yet modifying some more peripheral feature(s) of a person could leave (the central aspects of) her personality unaffected. Therefore, the directness can still be seen to make self-validating neuroenhancement—in which central aspects of a person’s self change—especially problematic. Accordingly, I henceforth treat the directness of neuroscientific techniques as the main reason for considering self-validating neuroenhancement incompatible with autonomy. In terms of the criteria of autonomy characterized above, the problem the directness is perceived to cause here relates to the requirements of understanding and intentionality: the mental changes brought about by neuroenhancement are not seen to result, *in an appropriate way*, from the knowing and intentional activity of the person undergoing them.

## Is the main reason for considering self-validating neuroenhancement heteronomous plausible?

Some neurointerventions work through thought processes and, hence, employ the rational capacities of the persons modified with the techniques, at least to an extent. A central example is neurofeedback, in which a person regulates her brain function on the basis of real-time neuroscientific displays of her brain activity. Transcranial current stimulation of medial prefrontal cortex, for another instance, is reported to enable the replacement of impulsive unconscious attitudes with rationally controlled reactions in a way that employs the rational faculties of the individuals undergoing the intervention (see, e.g., Diéguez and Véliz 2017; Sellaro et al. 2015). Assuming that these kind of techniques could be employed in connection with self-validating neuroenhancement, the view that such enhancement conflicts with autonomy because of the directness of the techniques it employs might not apply to all self-validating neuroenhancement. But, for the sake of argument, let us now focus on neurointerventions that are entirely direct. Would its employing such techniques be a sufficient reason to consider self-validating neuroenhancement incompatible with autonomy?

The answer to this question would appear to be no. To see why, consider Larry’s case. A few years ago, Larry too wanted to become more creative, cognitively able, and cheerful. He had learned that particular kind of nutrition—foods including omega-3 fatty acids, folic acid, choline, and iron, for instance,—physical exercise, and sufficient sleep can improve one in those respects (see, e.g., Dresler et al. 2013; Misuraca et al. 2017; Pilcher and Huffcutt 1996; Ratcliff and van Dongen 2009; Strasser and Fuchs 2015). Accordingly, Larry decided to dump his couch potato ways. He replaced his diet of junk food with healthy nourishment, started jogging, doing yoga, and going to the gym, and became careful to sleep at least 8 h each night. After that had went on for several months, Larry had become more creative, cognitively able, and cheerful than he was before. While he initially thought that he would ultimately remain the same old Larry his self-improvement notwithstanding, he also realized that his personality had changed in unexpected ways. He saw the world and himself differently than he used to do and wanted to behave in ways he did not expect to want before his life-style reform. Yet, because his enhancement process changed him, Larry accepted his new self without reservations.

Instead of having come into being through rational reflection on his part, Larry’s new personality came about as a result of the neurobiological changes that his adoption of the new lifestyle caused in him. Therefore, also Larry’s endorsement of his new personality is based on mechanisms that circumvented his rational capacities. Yet I take it that Larry’s approval of his new personality would commonly be deemed autonomous (see also, e.g., Bublitz and Merkel 2009, p. 367; Levy 2007, pp. 106–107). The intuition is buttressed by the following considerations. To begin with, Larry is acquainted with both his old and new self and understands that his personality change was grounded on the neurobiological alterations his new lifestyle caused. Indeed, before he realized that his self had changed in unexpected ways, Larry was not much interested in understanding how one becomes the kind of person one is. Accordingly, he may be even better aware of the origins of his new self than he is about the origins of his old self. Moreover, Larry’s personality change and his endorsement of his new self does not result from control by others. Hence, his acceptance of his new personality apparently meets the criteria of autonomy understood as “self-rule that is free from both controlling interference by others and limitations that prevent meaningful choice, such as inadequate understanding” (Beauchamp and Childress 2013, p. 101). Insisting that Larry’s endorsement of his new self cannot be autonomous because his personality change did not came about through rational reflection would just beg the question why autonomy as it is now understood would presuppose rational reflection in that sense. Is Kelly’s case then different from Larry’s in terms of the directness of the personality changes the individuals underwent?

It has been maintained that it is implausible to argue for A by maintaining that it is relevantly similar to some allegedly commonly accepted B when the acceptability of B is merely assumed (see, e.g., Mizrahi 2014). The position is plausible when intuitions about the acceptability of B in fact are rather unclear or vary significantly. Consider, for instance, intuitions related to the case in which you wake up in the morning to find yourself attached to a world-famous violinist whose circulatory system has been plugged into yours so that his life now depends on you (Thomson 1971, pp. 48–49). Yet the view that Larry’s endorsement of his new personality is autonomous is plausibly not overly unclear or controversial. The other main objection to arguments of the just described kind is that there after all is some important difference(s) between the A and B compared with each other. Accordingly, below I do my best to account for all of the features in which the cases of Kelly and Larry could now be deemed relevantly different from each other.

To begin with, Larry is male whereas Kelly is female, Larry is two years younger than Kelly, and Larry’s personality change took place earlier and in the town in which the couple resided previously whereas Kelly was enhanced more recently and in the city they live in now. Yet, other things being equal, such facts do not entail that Kelly’s change of self was more direct than Larry’s.

Besides in the terms just mentioned—and in the respect that Larry changed his lifestyle whereas Kelly was improved by neuroscientific means,—the personality changes of Kelly and Larry differ in that his was slower and more gradual than hers. It might be taken that slower and more gradual changes are more amenable to control by the person undergoing them. Yet slow and gradual changes can also be more difficult for the person to notice than fast and abrupt ones. And, as it happened, Larry focused on the anticipated alterations in creativity, cognitive ability, and mood and the actual realization that he is not the same old Larry anymore came only after that change had already occurred. Accordingly, that Larry’s change of self was slower and more gradual than Kelly’s does not make Larry’s change of self less direct than Kelly’s.

As Larry needed to alter his diet, exercise regularly, and sleep more than he did previously whereas Kelly just underwent a relatively undemanding neuroscientific procedure, Larry’s change of self presupposed more activity on his part than Kelly’s personality change required from her. Yet, again, that does not mean that the changes in Larry’s personality came about in a less direct manner than did the alterations in Kelly’s personality. Perhaps Larry could return to his old self by, say, adopting his old habits? Yet, even if Kelly’s enhancement were irreversible—which it need not be—that would not mean that the changes in her personality occurred more directly than did the alterations in Larry’s self. As the cases of Kelly and Larry would not appear to differ from each other in terms of the directness of the personality changes the individuals underwent, it would seem that Kelly’s endorsement of her new personality should be considered autonomous too.

## Further reasons for considering self-validating neuroenhancement incompatible with autonomy?

Yet the reasons considered above are evidently not the only possible reasons for thinking that self-validating neuroenhancement conflicts with autonomy. Below I briefly consider further possible grounds for deeming the kind of enhancement heteronomous.

### Neuroenhancement and freedom to fall

It has been argued that making people morally better by using neurobiological means—moral bioenhancement—would be detrimental to personal autonomy, because such improvement would deprive the enhanced people of “freedom to fall.” In this view, autonomy presupposes adequate opportunity to do wrong. Given that an agent who has undergone moral bioenhancement would lack adequate opportunity to do wrong, her autonomy would be compromised (Harris 2011; see also e.g. Hauskeller 2017). Someone might now maintain that the argument applies in Kelly’s case as well. As, or to the extent that, the enhanced Kelly lacks “freedom to fall,” the argument could proceed, she does not have adequate possibility to choose wrongly. Accordingly, the conclusion of the argument could be, Kelly’s endorsement of her new self cannot be autonomous.

However, as has been argued before, the line of argument appears unconvincing (see, e.g., Carter and Pritchard 2019; Danaher 2018; Diéguez and Véliz 2017; Savulescu et al. 2014). Assume that John, a person like the old Kelly, improves himself. The means he employs are not direct. Instead, John uses his rational capacities only. As a result of the enhancement, John becomes as creative, cognitively able, and cheerful as the new Kelly. Moreover, his personality changes in the same ways as hers. It would, I take it, be clearly counterintuitive to maintain that, because his ability to make wrong choices is now lesser than it was before the enhancement process he underwent, John’s endorsement of his new self cannot be autonomous. Accordingly, given that both Kelly and John are equally unable to choose wrongly, her lacking “freedom to fall” would not appear to compromise the autonomy of Kelly’s endorsement of her new self. Hence, if Kelly’s neuroenhancement makes her endorsement of her new self heteronomous, the reason apparently must lie elsewhere. But where? The central difference between John and Kelly is that Kelly’s enhancement process employed direct means whereas John’s did not. Yet, the idea that the directness of neurointerventions makes neuroenhancement incompatible with autonomy was already discussed above. Insofar as it is accepted that Larry’s endorsement of his new self qualifies as autonomous, as I proposed that it does, this possible objection should be rejected.

### A problem of inauthenticity?

The notion of authenticity is sometimes seen to be intimately related to autonomy and has also been discussed in connection with neuroenhancement (see, e.g., Levy 2007, 2011). Accordingly, someone might now maintain that authenticity is an important element of autonomy that should be duly acknowledged in assessing whether Kelly’s acceptance of her new self qualifies as autonomous. Indeed, a critic might argue that the new self Kelly acquired within the neuroenhancement process she underwent is inauthentic. And for that reason, the conclusion of the critic could be, Kelly’s endorsement of her new self cannot be autonomous. In this view, the alleged inauthenticity of Kelly’s new self thus entails that her acceptance of her new personality must be heteronomous.

However, it is not clear how authenticity should be understood: philosophical literature on authenticity is “deeply fraught and riddled with controversy” (Rings 2017, p. 475). As things are, the senses philosophers have given to the notion vary from loyalty to one’s essence to self-creation to honest self-presentation to self-fulfillment to reflective endorsement of one’s self, to mention the central examples (cf., e.g., Bauer 2017; Kadlac 2017; Rings 2017). Whether a person modified by neuroscientific means presents her personality honestly, for instance, can plausibly be relevant in assessing her in moral terms (Kadlac 2017; see also, e.g., Schermer 2017). Yet it is unclear why honest self-presentation, loyalty to one’s essence, self-creation, or self-fulfillment should be seen as preconditions of *autonomy* (see also, e.g., Oshana 2007).^14^ Why, for instance, could one not be autonomous in wanting to improve the undesirable features that one’s essence might involve?^15^

It has been proposed that choices unaffected by a neurointervention are at least more autonomous than choices affected by such an intervention, because the former are based on a person’s “natural self rather than on his stimulation-influenced self” (Müller and Walter 2010, p. 211). In this view, choices unaffected by neurointerventions are natural whereas choices affected by neuroscientific means are not, because the latter are “caused by a technical device built into the brain, not by the brain itself” (Müller and Walter 2010, p. 211). However, neurointerventions would assumedly typically rather just affect the functioning of an individual’s brain—say, modulate the activity of certain neural circuits—than replace it by a technical device that then decides on the individual’s behalf. Insofar as the problem referred to here relates to the directness of neuroscientific techniques, it was already considered above.

Requiring that an individual reflectively endorses his new self does seem important from the viewpoint of autonomy, at least when the individual’s personality has suddenly changed in some important and unforeseen way(s). Yet, as explained, Kelly knowingly approves of her new self. And with her new capacities, she may well be even more able to reflect upon her personality than she was before her enhancement process.^16^ That she needs to knowingly approve of her new self to be self-governing with respect to it would also appear to be already presupposed by the criteria of autonomy characterized above (see Sect. “Main starting points”). For it would seem that otherwise Kelly’s having the new personality could not accord with her own desires and plans. Accordingly, considerations of authenticity would not appear to entail that Kelly’s self-validating neuroenhancement cannot be autonomous.

### Misuses, abuses, and disordered mental conditions

Others might pressure, manipulate, or even coerce a person to engage in neuroenhancement, so as to, say, make him a more efficient worker (see, e.g., Appel 2008). Neuroenhancement might perhaps also employ technology through which others could—either intentionally or unintentionally—affect an enhanced person or her behavior in some way(s) he would not agree with (cf., e.g., Pugh et al. 2018). And some neurointerventions are reported to sometimes induce mania and states reminiscent of dissociative identity disorder (see, e.g., Klaming and Haselager 2013). Accordingly, someone might propose that these kind of problems related to neurointerventions imply that Kelly’s endorsement of her new self is heteronomous.

An individual’s endorsement of the new personality he acquires in self-validating neuroenhancement that involves the kind of problems just described above may well be heteronomous. Yet the problems would not seem to be essential or inseparable features of self-validating neuroenhancement, to say the least. How often neuroenhancement techniques would be misused or abused in connection with self-validating neuroenhancement and how frequently such enhancement would involve disordered mental conditions are empirical questions that I unfortunately am unable to answer. But insofar as self-validating neuroenhancement is not shown to be especially likely to involve such problems, the difficulties are not sufficient reasons for concluding that self-validating neuroenhancement is especially likely to be heteronomous. That such problems may sometimes occur in its connection definitely does not show that self-validating neuroenhancement cannot be autonomous.

## Conclusion

In what I called self-validating neuroenhancement, the enhanced individual’s personality changes in significant way(s) she did not foresee and the individual endorses her new self because the neurointervention changed her. Above I considered whether self-validating neuroenhancement can be autonomous. I focused on what has been deemed a/the central understanding of individual autonomy in contemporary bioethics (and in applied ethics more generally). I assessed several possible grounds for considering self-validating neuroenhancement incompatible with autonomy and maintained that they do not entail that self-validating neuroenhancement cannot be autonomous. The above considerations do not show that self-validating neuroenhancement is morally unproblematic nor that it cannot undermine autonomy (see, e.g., Sect. “Misuses, abuses, and disordered mental conditions”). Yet, insofar as the considerations are adequate, self-validating neuroenhancement can be autonomous.

Given that her decision to improve herself was not unduly affected by others and that her enhancement process did not result in mental disorder, the conclusion suggests that Kelly’s endorsement of her new personality is autonomous. The conclusion also implies that neuroenhancement likely to be self-validating need not be avoided out of the fear that such enhancement necessarily results in heteronomy. In light of the above considerations, the fear is misguided. As proposed, the conclusion that self-validating neuroenhancement can be autonomous applies, *mutatis mutandis*, to the cases of self-invalidating and indecisive neuroenhancement as well.^17^ Besides self-validating, self-invalidating, and indecisive neuroenhancement, the conclusion pertains to corresponding forms of neuroscientific treatment too (see note 12) and can also be of relevance in assessing at least some forms of, say, genetic treatment and enhancement.

## Footnotes

1 In moral philosophical literature, individuals whose personalities are, in some sense, replaced by new ones have mainly been discussed in psychiatric ethics and in connection with medical advance directives (see, e.g., Haji 1997; Dworkin 1993, Chap. 8). Yet the cases focused on in those debates would not appear to be similar enough to self-validating neuroenhancement to throw sufficient light on the question whether such enhancement can be autonomous. Witt (2017) touches on self-validation in connection with discussing autonomy and neuroscientific treatment. Insofar as it pertains to the topic of this article, his view will be considered below.

2 If self-validating neuroenhancement cannot be autonomous, it might be employed, say, to improve the mood of a person who has permanently lost her autonomy or will shortly lose it for good. In the latter case, the person might choose to undergo a neurointervention foreseen to be self-validating, while he is still autonomous. Then the person could be said to be autonomous with respect to the intervention, even though his subsequent endorsement of his new self were heteronomous. I now put such cases aside.

3 The technology employed in enhancing human beings could assumedly be used in improving some other beings too. Yet humans are the primary—although not necessarily the only (cf., e.g., Beauchamp and Wobber 2014)—examples of autonomous beings and I am better informed about humans than about other beings (in whose case questions about endorsement of selves might not arise at all). Accordingly, I now focus on humans. Needless to say, the idea that human capacities should sometimes be enhanced is not new. For a long time, people have used caffeine to improve alertness, chocolate to better mood, and physical exercise to develop fitness, to mention just a few examples.

4 Neuroenhancement of physical capacities and neuroscientific lifespan extension would appear to be different enough from neuroenhancement of mental abilities to merit their own discussion.

5 If his fully knowing what it is like to be the new Mike presupposes his actually acquiring the new personality, Mike’s case too would involve some initially unforeseen consequences. Yet Mike’s post-enhancement endorsement of his new personality could be taken to result from his having been altered by neuroscientific techniques, even if his case did not involve unpredicted consequences.

6 I now put aside the question whether enhancement is the best term to use in connection with what I named self-invalidating and indecisive neuroenhancement.

7 For responses to central objections to the conception of autonomy see, e.g., Beauchamp (2009) and Beauchamp and Childress (2013). Some of its critics rather want to further specify the conception or to clarify the material, social, and communicative preconditions that should be met to attain autonomy in that sense than to reject it (cf., e.g., Jennings 2016).

8 Accordingly, the conception of individual autonomy is also commonly seen to ground the practice of medical informed consent, in which the moral acceptability of a person’s undergoing a medical intervention is made contingent on her autonomously authorizing the intervention (see, e.g., Beauchamp 2009, p. 58; Beauchamp and Childress 2013, Chap. 4).

9 Conceptions about the nature of individual autonomy vary widely (see, e.g., Dworkin 1988; Garnett 2014a, b; Korsgaard 2009) enough to suggest that autonomy theorists are not always focusing on the same notion. Accordingly, noting that attempts to find a core essence for autonomy have not been successful, Anderson, for instance, argues that normative debates about criteria for autonomy are best conceived as debates about “the relative merits of various possible packages of thresholds, entitlements, regulations, values, and institutions” (Anderson 2014, p. 355), so that autonomy can mean different things in different contexts.

10 For discussion on other problems neuroenhancement confronts see, e.g., Jotterand and Dubljevic (2016), Levy (2007), Savulescu and Bostrom (2009), and Savulescu et al. (2011).

11 Someone might propose that Kelly’s endorsement of her new personality cannot be autonomous insofar as the old Kelly would not approve of the new Kelly. But why should the autonomy of the new Kelly depend on her being approved by the old Kelly? After all, that the self I had before would disapprove of me as I now am does not entail that my current personality is heteronomous (see also, e.g., Juth 2011, p. 43).

12 See also, e.g., Müller and Walter (2010) and Focquaert and Schermer (2015). Witt concentrates on treatment but proposes that what he says applies to enhancement as well (see Witt 2017, p. 387). Indeed, while some clear cases of both treatment and enhancement exist, the division between treatment and enhancement appears difficult to uphold in general (see, e.g., Levy 2007, Chap. 3). Witt refers to the account of autonomy presented by Beauchamp and Childress (Witt 2017, p. 387), but wants to specify it in the way proposed in the above quotation. He (2017, pp. 389–390) goes on to argue that a patient’s approval of an identity-changing intervention is an informed consent only if the approval is based on an assessment of her pre-intervention quality of life and the identity change from her pre-intervention perspective and an assessment of her post-intervention quality of life from her post-intervention perspective, provided that pre-intervention and post-intervention quality of life are weighted equally and the weight assigned to the identity change is that which the patient endorses when considering her weighing preferences from her pre-intervention perspective. If the reason for looking at the personality change a person undergoes within a neurointervention from the perspective of the person’s old self referred to here is plausible, the problem focused on in this article—whether self-validating neuroenhancement conflicts with autonomy—is solved. Accordingly, I now concentrate on that reason.

13 The same applies in the cases of several of the other difficulties of self-validating neuroenhancement discussed in this article. Yet, as proposed, previous discussions on the difficulties have not focused on self-validating neurointerventions.

14 To clarify, I am not saying that those who understand authenticity in one or more of the senses referred to above necessarily see authenticity as an element of autonomy.

15 Kelly’s new capacities can also improve her ability to be loyal to her essence or to create herself (see also, e.g., Levy 2011) and the new Kelly need not be less honest in her self-presentation or less self-fulfilled than the old Kelly, if these kind of things were relevant to autonomy.

16 Reflective endorsement of one’s personality can, of course, be understood in some other sense than the one referred to above. But then it should be clarified what the other sense is and why autonomy should presuppose reflective endorsement in that sense.

17 In the case of reversible self-invalidating neuroenhancement, for example, the result speaks for returning to an enhanced individual’s old self rather than for his trying to come to terms with his new self. In the case of indecisive neuroenhancement, the conclusion implies, not that indecisiveness itself is cannot be a problem from the viewpoint of autonomy, but that the significance of the indecisiveness related to that kind of enhancement rationally cannot be downplayed as not reflecting the autonomous views of the enhanced person in question.
